# Chronic pain after groin hernia repair: pain characteristics and impact on quality of life

**DOI:** 10.1186/s12893-020-00805-9

**Published:** 2020-07-06

**Authors:** David Bande, Luis Moltó, Jose Antonio Pereira, Antonio Montes

**Affiliations:** 1 Pain Clínic, Department of Anaesthesiology, Institut Municipal d’Investigació Médica. Parc de Salut Mar, Universitat Autònoma de Barcelona, Passeig Martím 25–29, 08003 Barcelona, Spain; 2 General Surgery Department, Institut Municipal d’Investigació Médica. Parc de Salut Mar, Departament de Ciéncies Experimentals i de la Salut (CEXS), Universitat Pompeu Fabra, Barcelona, Spain

**Keywords:** Chronic pain, Hernia repair, Quality o life

## Abstract

**Background:**

Chronic postsurgical pain (CPSP) after hernia repair research has mainly relied on unconfirmed self-reporting. We aimed to describe confirmed CPSP incidence, management, and quality of life (QoL) in a 2-year prospective study.

**Methods:**

Multicenter study (GENDOLCAT) of 3890 patients undergoing 4 common surgical procedures in 23 hospitals to develop a risk model for CPSP; 2352 men underwent open hernia repair. Patients with pain were identified by telephone at 1 and 3 months and referred to the hospital 4 months after surgery for a physical examination to confirm CPSP. Three validated tools were used: the Brief Pain Inventory (BPI) for severity, analgesic use, and interference with activities; the SF-12 questionnaire for QoL (validated Spanish version), and the Douleur Neuropathique 4 (DN4). Patients with CPSP were called again at 1 and 2 years.

**Results:**

In 1761 patients who underwent hernia repair and were eligible for physical examination for CPSP, the incidence of confirmed pain at 4 months was 13.6% (patient-reported pain, 6.2% at 1 year and 4.0% at 2 years). Neuropathic pain was diagnosed in 38.5% of the CPSP patients at 4 months. The incidences of neuropathic CPSP in patients with mesh or non-mesh repairs were similar (38.6 and 33.3%, respectively). SF-12 physical component scores changed little in all patients, whether or not they developed CPSP. The SF-12 mental component decreased significantly in all patients, but the decrease was clinically significant only in CPSP patients. CPSP interfered with activities (18%), work (15.6%), walking (15%) and mood (10.2%). At 2 years 52.1% of CPSP patients had moderate/intense pain and 28.2% took analgesics.

**Conclusion:**

CPSP affects QoL-related activities, and although it diminishes over the course of 2 years after surgery, many patients continue to have moderate/intense pain and take analgesics. CPSP and neuropathic pain rates seem to be similar after mesh and non-mesh repair. BPI and SF-12 mental component scores detect effects on QoL.

**Trial registration:**

ClinicalTrials.gov NCT01510496.

## Background

Chronic postsurgical pain (CPSP) is a frequent complication in the general surgical population [1, 2]. After open groin hernia repair, a procedure undergone by more than 20 million patients every year [3], the reported incidence of CPSP ranges widely from 6.9 to 60% [1, 4–6]. The reason for discrepancies is thought to be the differing research designs applied, especially regarding diagnostic criteria and the means of gathering evidence, by postal questionnaires [2, 7, 8] or telephone interviews [9, 10], for example, rather than by physical examination as used in some studies [1, 3].

The features and persistence of CPSP and its impact on quality of life (QoL) have been reported after studies seeking to establish risk factors [1–3, 11–13]. Most groups, however, have not included long-term follow-up of patients. Nor have they diagnosed or determined the characteristics of CPSP by means of physical examination. The population-based GENDOLCAT study, which gathered data on CPSP for 2 years in four surgical scenarios, included a large prospective cohort of 2352 men undergoing hernia repair [1]. Designated anaesthesiologists in each centre completed preoperative clinical questionnaires according to protocol. A telephone interview to screen for possible CPSP was done 3 months after surgery, and patients reporting pain were given a hospital outpatient appointment at 4 months to confirm CPSP. CPSP developed in 13.6% after hernia repair and persisted up to 2 years in a sizeable minority. Although no genetic differences were identified in CPSP patients, a risk model based on presurgical clinical variables alone identified 73% of the cases. The six predictors were type of surgery, younger age, general physical and mental health status (SF-12), and prior pain at the site of surgery or another area. The study generated extensive information about the course of confirmed CPSP, including the distinction between neuropathic and non-neuropathic pain, but data for specific surgeries was only partially reported. A detailed understanding of the course of pain after hernia repair has the potential to inform surgeons and pain physicians who follow patients with CPSP as well as researchers interested in exploring the effectiveness of different management approaches.

The aim of the present analysis of the GENDOLCAT data for physician-confirmed diagnoses of CPSP after hernia repair was to include full descriptive information on clinical features and management of pain not reported earlier, with particular focus on neuropathic pain. We discuss pain features in the context of incidence rates, interference with QoL at 4 months (the main GENDOLCAT outcomes), and the course of pain over the full 2-year period.

## Methods

The prospective, multicentre, population-based GENDOLCAT study of clinical and genetic risk factors for CPSP in 23 hospitals in Catalonia and Valencia between 2009 and 2010 [1]. The open groin hernia repair cohort included 2352 male patients (code 53.00, 53.05, 53.10, 53.16, 53.17, 53.29, 53.9, International Classification of Diseases, 9th Revision) who were operated under general or regional anaesthesia or local anaesthesia with sedation. Women were not enrolled in the hernia group to rule out sex as a confounding factor in the study of genetic risk, which the GENDOLCAT study explored. Patients were excluded if they were under the age of 18 years, required reintervention or were scheduled for endoscopic surgery (no incision), or had a significant mental disorder. In addition, relatives (parents, grandparents, children, grandchildren, or siblings) who had already been included in one of the other surgical groups were excluded, given that their inclusion could have skewed the study of gene associations that formed part of the wider GENDOLCAT aims. The study was approved by the clinical research ethics committee of the leading centre, Parc de Salut Mar (file reference CEIC-IMAS: 2008/3080/I) and the commitees of all other centres. Patients signed informed consent statements for data collection and use and agreed to follow-up telephone interviews and an outpatient hospital visit, if necessary, to confirm CPSP by physical examination. The study was registered before data collection began (ClinicalTrials.gov NCT01510496).

### Outcomes and data collection

Patients who might have CPSP were identified in two preliminary telephone interviews between the first and third months after surgery. If pain was reported, they were given appointments for a physical examination and clinical interview. CPSP was defined by a modified version of the diagnostic criteria of Macrae and Davies [14]: 1) pain developed after a surgical procedure, 2) pain lasted at least 3 months after surgery, 3) no other causes, such as cancer or chronic infection, could be found to explain the pain, and 4) absence of the same pain (location or sensation) before surgery. The main outcomes at approximately 4 months after groin hernia repair were the characteristics of CPSP and the degree to which pain at that time interfered with daily living and its effects on QoL. We were particularly interested in identifying whether or not pain was neuropathic at this time and recording the features and the locations of neuropathic and non-neuropathic pain. Secondary outcomes were changes in the incidence of reported CPSP up to approximately 2 years after surgery and reported approaches used to manage pain and their effectiveness.

Each hospital centre recruited a researcher or research team. One anaesthesiologist from each team was designated to attend training sessions on how to gather data before surgery and during hospitalisation, and to diagnose CPSP and neuropathic pain at 4 months. The data manager (J. Cantillo) conducted all the follow-up telephone interviews. (See Online Resource 1 for questionnaires and clinical definitions.)

Before surgery, a trained anaesthesiologist administered the validated Spanish version [15] of the Hospital Anxiety and Depression Scale (HADS), which has proven useful for diagnosing or ruling out anxiety or depression in patients without a prior history of psychiatric problems. The Short Form Health Survey-12 questionnaire (SF-12, Spanish version 2 [16]) was used to assess the physical and mental components of QoL. Also recorded at this time were physical status according to the American Society of Anesthesiologists’ classification; prior pain in the area of surgery and pain in other parts of the body expressed on a verbal numerical rating scale of 0 to 10 (0 = no pain, 10 = the worst imaginable pain) and a verbal categorical pain scale (no pain or mild, moderate, intense, or unbearable pain); history of treatment with analgesics; concomitant diseases; and any history of substance addiction to street drugs, alcohol, or smoking.

During and after surgery we recorded the following variables: duration of surgery, duration of hospital stay, anaesthetic technique used, whether or not techniques were used to spare nerves in the surgical field (the iliohypogastric and ilioinguinal nerves, and the genital branch of the genitofemoral nerve), opioid use, and intraoperative complications. Postoperative pain on the two scales and analgesia used were recorded for 24 h after wound closure.

The next phase of data collection involved the standardised telephone questionnaire (see Online Resource 1). In the first two interviews at approximately 1 and 3 months after surgery, the researchers sought to identify patients to ask about continued pain. Those who reported such pain at the second phone call were given outpatient clinic appointments for an exhaustive physical examination to confirm the diagnosis of CPSP. During the diagnostic examination, the examiner, who was an anaesthesiologist expert in pain management, used the following assessment instruments: the Brief Pain Inventory (BPI), which records pain severity, analgesic use, and interference with daily living, the Spanish SF-12 questionnaire, and the Douleur Neuropathique 4 (DN4) inventory to detect neuropathic pain [17]. The BPI rated pain interference on a scale of 0 to 10 (0 = interfered not at all; 10 = interfered completely), and a score of 3 or more was defined as clinically important. A positive response to 4 out of 10 items on the DN4 classified pain as neuropathic. As part of the DN4 assessment, the examiner tested for hypoesthesia (light touch with a cotton swab, pinpricking with Von Frey filaments) and dynamic allodynia (brushing). The exact location of pain (upper or lower abdomen, scar, groin, thigh, external genitals) was also recorded. Both the operated and unoperated sides were examined. We also asked the patient to report the use of specific analgesics on a list (see Online Resource 1) so that type and combinations were on record. Patients who reported no pain during the first and second telephone interviews were sent an SF-12 questionnaire by ordinary post and asked to fill it out and return it in an envelope provided. Patients whose diagnosis of CPSP was confirmed by physical examination were called again at approximately 1 year and, if pain had persisted, they were also contacted at approximately 2 years. During these calls the interviewer filled in a standardised questionnaire to evaluate pain intensity, whether the pain was continuous or intermittent, analgesic use, and whether or not the patient had returned to work. The National Health Service Death Register was checked for exitus if patients were not reached.

To evaluate the quality of data collection, four authors audited the medical records of a random sample of 5% of the patients from 5 randomly chosen centres. The total number of audits was 22. A total of 22 instances of error in a variable or missing data were found, giving an error rate of 1.2% of the data audited.

### Statistical analysis

We compiled descriptive statistics for all demographic and clinical variables. Clinical variables included a finding of neuropathic pain (DN4), use of postoperative analgesics, use or non-use of nerve-sparing techniques, BPI results and scores on physical and mental components of the SF-12. Continuous variables were presented as mean (SD). Categorical variables were presented as number of observations and percentage of the relevant number of cases with available data. SF-12 results at 4 months were compared with the *t* test (if paired and normally distributed) or the Wilcoxon test (if unpaired and non-normally distributed). The CPSP incidence was calculated for the population of included patients, after removing early exclusions (during hospitalization), withdrawals, deaths, and patients not located after 2 phone calls. Patients lost during telephone screening before the 4-month physical examination were counted as having no CPSP. This decision was made in order to avoid overestimating the percentage of chronification. Cases were classified as CPSP on the basis of confirmed presence of pain related to surgery in the follow-up visit at the patient’s hospital.

## Results

**Fig. 1:**
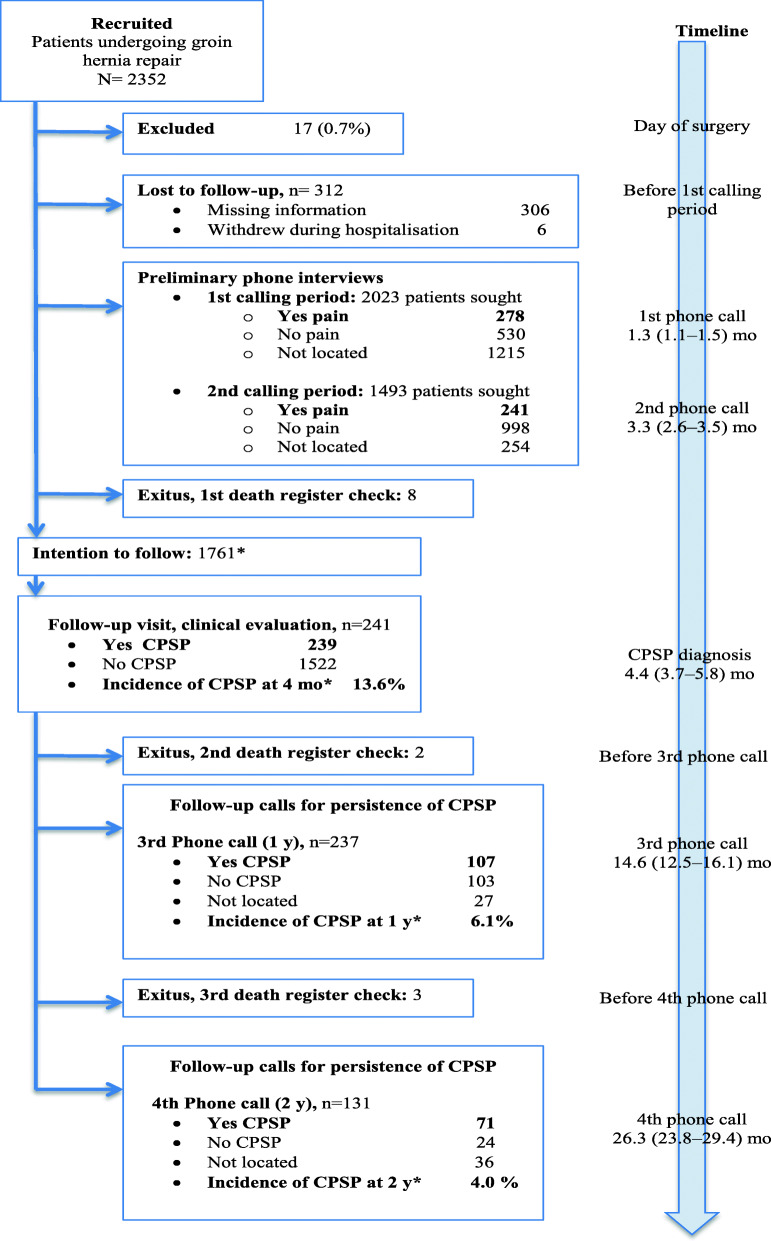
Flow chart showing patients recruited when scheduled for groin hernia repair, those lost before the telephone interviews, and the group of 239 patients initially diagnosed with CPSP based on physical examination and followed for 2 years. ^a^In order not to overestimate the frequency of CPSP, incidence rates were calculated on a cohort of 1761 patients we intended to follow: from the 2352 patients recruited, 17 were excluded as having been inappropriately enrolled, 312 were lost before phone calling started at 1 month, an additional 254 could not be reached by phone before the diagnostic examination at 4 months, and 8 deaths had occurred. The timeline shows median (10–90th percentile) times in months when data collection occurred, counting from the day of surgery

Of the 2352 patients recruited, 1761 were located and eligible for assessment for CPSP at 4 months, after 591 losses (25.1%) as shown in the flow chart *(*Fig. 1). Patient, surgical, and anaesthetic characteristics in this cohort are detailed in Table 1. The presence of CPSP was confirmed in 239 (13.6%) during the DN4-guided physical examination at 4 months, excluding 2 patients whose pain was caused by another disease; 13.9% of those undergoing mesh repairs (236/1693 patients) developed CPSP versus 4.4% (3/68 patients) of those repaired without mesh, although the difference did not reach statistical significance (*P* = 0.053). Of the 1522 patients who reported no pain in the first and second telephone interviews and who were sent the postal questionnaire (SF-12), 471 (30.9%) responded.

**Table 1:** Characteristics of the cohort of 1761 patients^a^

Age, years, mean (SD)	59.01 (14.02)
**BMI, kg/m**^**2**^**, mean (SD)**	26.11 (3.26)
**ASA physical status,*n/N*(%)**	
1. normal healthy patient	543/1761 (30.8)
2. patient with mild systemic disease	1027/1761 (58.3)
3. patient with severe systemic disease	187/1761 (10.6)
4. patient with severe systemic disease that is a constant threat to life	4/1761 (0.2)
**Anxiety, HADS,*n/N*(%)**	318/1705 (18.7)
**Depression, HADS,*n/N*(%)**	108/1705 (6.3)
**Duration of surgery, min, mean (SD)**	45.57 (21.90)
**Meshless hernia repair**	68/1755 (3.87)
**Anaesthetic technique,*n/N*(%)**	
General	241/1736 (13.9)
Combined (general + local/regional)	138/1736 (7.9)
Neuroaxial	1296/1736 (74.6)
Ilioinguinal, abdominal-genital, or transversus abdominis plane blockade	12/1736 (0.7)
Local anaesthetic infiltration	49/1736 (2.8)
**Endovenous opioids during surgery,*n/N*(%)**	672/1733 (38.8)
**Complications during surgery,*n/N*(%)**	20/1734 (1.2)
**Prior analgesic treatments,*n/N*(%)**	397/1752 (22.7)
**Diagnosed chronic illness,*n/N*(%)**	230/1756 (13.1)
**Street drug dependence,*n/N*(%)**	29/1755 (1.6)
**Alcohol dependence,*n/N*(%)**	252/1756 (14.4)
**Smoking,*n/N*(%)**	
Smoker	486/1756 (27.7)
Ex-smoker	388/1756 (22.1)
**Non-use of nerve-sparing techniques,*n/N*(%)**	312/1756 (17.8)
**Hospital stay, days, mean (SD)**	0.61 (1.54)

^a^ The cohort of patients followed for chronic postsurgical pain after hernia repair, after exclusions and early losses (exitus, missing information before pain screening started, withdrawals) (*Fig*. 1). Numbers shown in denominators in the table reflect the total number of patients (*N*) for whom data were available for the variable named

**Fig. 2:**
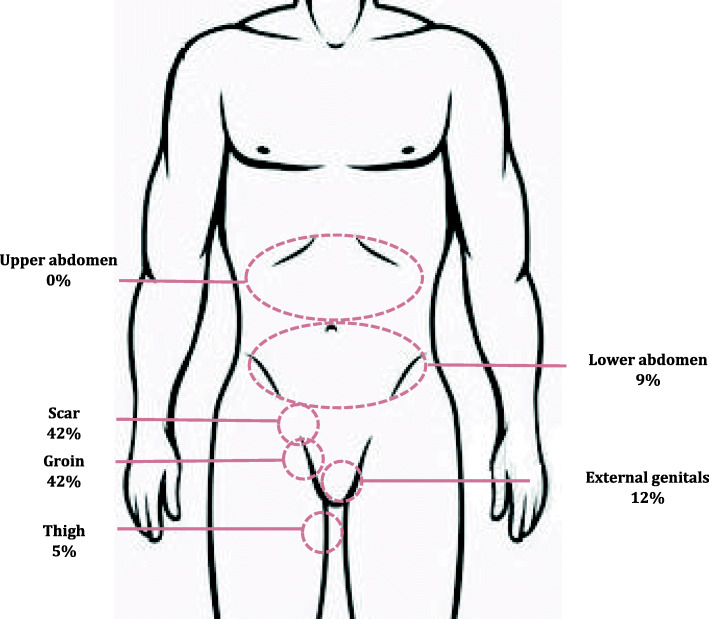
Locations of diagnosed CPSP at 4 months in 239 patients

Figure 2 shows the locations of diagnosed CPSP at 4 months. Pain was most often in the groin (42%) and at the scar (32%).

Neuropathic pain was diagnosed in 92 (38.5% of the 239 of the patients with confirmed CPSP). The difference in the rates of neuropathic pain between patients with mesh (38.6%, 91/236) and non-mesh repairs (33.3%, 1/3) was not significant. Table 2 shows the distribution of signs and symptoms of neuropathic pain.

**Table 2:** Neuropathic pain characteristics according to the self-report questionnaire portion of DN4 diagnostic tool

Characteristics	Patients, *n (%)*^a^
Burning, *n (%)*	25 (27.2)
Painful sensation of cold, *n (%)*	11 (6.5)
Electric shocks, *n (%)*	27 (29.3)
Tingling, *n (%)*	33 (35.9)
Pins and needles, *n (%)*	43 (46.7)
Numbness, *n (%)*	44 (47.8)
Itching, *n (%)*	24 (26.1)
Hypoesthesia to touch, *n (%)*	42 (45.7)
Hypoesthesia to prick, *n (%)*	30 (32.6)
Brushing, *n (%)*	13 (14.1)

^a^ A total of 92 patients with a DN4 score ≥ 4 (positive score on at least 4 items) were diagnosed with neuropathic chronic postsurgical pain. The percentages for each symptom are of the 92 patients

Table 3 summarises the life-interference effects of pain as shown by changes in the physical and mental component scores on the SF-12 questionnaire at 4 months for all patients with confirmed CPSP and the 471 patients without CPSP who responded to the postal questionnaire. Physical component scores were similar in patients with and without CPSP both before and after hernia repair. Although scores on the mental component decreased significantly from baseline in both groups, the decrease was clinically significant (≥ 3 points) only in patients confirmed to have CPSP. Thirty of the 167 patients with CPSP who also fully completed the BPI questionnaire (18%) reported that pain interfered with their general activities at 4 months. The reported interferences involved walking (25 [15%]), enjoyment of life (19 [11.4%]), work inside or outside the home (26 [15.6%]), mood (17 [10.2%]), personal relationships (15 [9%]), and sleep (7 [4.2%]. Three of those who had not reached the age of retirement (1.3%) had nevertheless not yet returned to work 2 years after surgery.

**Table 3:** Effect of pain on QOL at 4 months: changes in SF-12 scores, mental and physical components, for patients with and without CPSP

CPSP	SF-12	Presurgical		4 mo		Difference		*p* value^a^
		Mean	(SD)	Mean	(SD)	Mean	(SD)	
Yes	Physical component	42.81	(9.72)	41.10	(10.68)	−1.71	(10.09)	0.266
	Mental component	54.00	(8.58)	50.92	(10.80)	−3.08	(8.95)	0.028
No^b^	Physical component	48.37	(8.45)	48.39	(8.53)	0.03	(9.27)	0.967
	Mental component	55.96	(8.11)	54.22	(9.19)	−1.65	(9.34)	0.001

^a^ SF-12 results were compared with the *t* test for normally distributed paired results when CPSP was present or the Wilcoxon test when CPSP was not present

^b^ Although all patients with CPSP completed the SF-12, the postal response rate for non-CPSP patients was only 30.9%

Four months after surgery 32 patients with CPSP (18.9%) reported taking nonsteroidal anti-inflammatory drugs or paracetamol, 2 (1.2%) took weak or strong opioids, and 5 (3.0%) took adjuvant treatments (anticonvulsant agents or antidepressants) (Table 4). These figures had changed little 1 and 2 years after surgery. Thirty percent of these patients taking medication for CPSP felt their treatments relieved pain adequately.

**Table 4:** Use of pain medications by patients with CPSP

	4 mo ***N = 169***	1 y ***N = 103***	2 y ***N = 71***
**Any pain medication,*n (%)***	42 (24.9)	28 (27.2)	20 (28.2)
**Anti-inflammatory and/or paracetamol,*n (%)***	32 (18.9)	23 (22.3)	18 (25.4)
**Weak opioid without paracetamol,*n (%)***	2 (1.2)	0 (0)	1 (1.4)
**Strong opioid,*n (%)***	0 (0)	1 (1)	1 (1.4)
**Anticonvulsant and/or antidepressant,*n (%)***	5 (3.0)	1 (1)	1 (1.4)
**Other**	12 (7.1)	9 (8.7)	4 (5.6)

The incidence of CPSP (Table 5) decreased over time from 13.6% at 4 months to 4.0% at 2 years. However, pain intensity remained high (4.2 on the numerical pain scale) in patients who still had CPSP at 2 years. Furthermore, over half of those with pain at 2 years (52.1%) described it as moderate or intense.

**Table 5:** Incidence and intensity of CPSP according to numerical and categorical pain scale results

	4 mo	1 y	2 y
**CPSP incidence,*n (%)***	239 (13.6)	107 (6.2)	71 (4.0)
**Verbal numerical pain scale, mean (SD)**	3.9 (2.0)	4 (2.1)	4.2 (2.0)
**Verbal categorical pain scale,*n (%)***			
Mild	128 (53.6)	51 (48.1)	34 (47.9)
Moderate	88 (36.8)	39 (35.8)	25 (35.2)
Intense/excruciating	23 (9.6)	17 (16.1)	12 (16.9)

## Discussion

This prospective population-based study found that 13.6% of the cohort of 1761 patients had firmly diagnosed CPSP at 4 months after groin hernia repair. The incidence of CPSP fell substantially by about two-thirds (to 4.0%) after 2 years. This study also provides exhaustive information on the location and characteristics of pain, particularly for the 38.5% of CPSP patients with neuropathic pain. We found that the interference of pain with QoL could be detected mainly by the mental component of the SF-12. Finally, even though the incidence of CPSP fell substantially over 2 years, the proportion of patients who continued to take analgesics remained stable at about a quarter of the CPSP patients. Nonetheless, only 30% of those still on analgesics reported they were effective.

The proportion of CPSP patients we diagnosed with neuropathic pain after hernia repair was higher than the 28.7% other authors identified by means of neuropathic pain descriptors in the short-form McGill Pain Questionnaire [18] but lower than the 64% identified as neuropathic based only on the descriptor “aching” in another study [19] and the 55% identified by a list of neuropathic descriptors that did not form part of a validated scale in a third study [20]. We attribute these discrepancies to differences in study design, specifically the instruments used. Few researchers evaluate this type of pain using a specific questionnaire that requires physical examination, such as the DN4. Rather, they rely exclusively on descriptors (such as aching, burning, cutting, stabbing) associated with neuropathy. Our results for pain location is also relevant for interpreting reported incidence rates of neuropathic pain that has not been diagnosed by physical examination. Most of our patients with CPSP reported pain in the groin (42%) and/or scar (32%), as in another study [21]. However, some also reported pain at unexpected locations, even ones that bear no apparent relation to the innervation of the surgical field. Nine percent, for example, reported pain in the lower abdomen. These findings underline the importance of careful physical examination in diagnosing CPSP and whether it is neuropathic or not, even when nerve damage from surgery is not suspected or a specific damaged nerve cannot be identified. Thus, pain detected in unexpected locations, while there is absence of pain in expected ones, does not rule out CPSP, which can develop not only as the result of evident nerve damage from surgery but also from an inflammatory response unrelated to neuropathy [11]. Inflammation arises with the nociceptive stimulus of the incision, after which pain may be exacerbated by the sensitisation of peripheral nerves and the central nervous system [22].

We assessed the interference of pain with QoL with two instruments, the SF-12 and the BPI. A decrease of 3.08 points was detected in the SF-12 mental component score at 4 months in patients with CPSP, an effect that was just over the threshold considered clinically important. The decrease in this measure in patients without CPSP, while statistically significant at 1.65 points, was well below that threshold. However, it is important to remember that the response rate in non-CPSP patients was only 30.9%, whereas all patients with CPSP responded to the SF-12. Physical component scores changed little after surgery in all patients, regardless of the presence or absence of diagnosed CPSP. We think that the presence of a mental, but not a physical, effect on QoL can be attributed to the unexpected nature of long-term pain. In other words, the physical impact of pain might be relatively minor but the fact that it takes a considerable number of patients by surprise exacerbates the emotional impact. If our interpretation is right, these results suggest that patients undergoing hernia repair procedures should be informed of the possibility of CPSP and given discharge instructions on how and when to seek care if pain persists beyond 3 months. Of the three other studies that used used the SF-12 to assess QoL after hernia repair [23–25], one of them [25] also reported that all patients, whether with or without CPSP, had significantly improved physical component scores, but not mental component scores, after 12 months. Our BPI results revealed that 18% of CPSP patients found that pain interfered with daily activities, 15% reporting that walking was affected. One systematic review found that 32% of patients reported that CPSP affected daily and leisure activities and/or work; however, only 2 of the 7 studies reviewed used a validated instrument (the SF-36) [4].

A large number of validated instruments are available to assess QoL and function: the Activities Assessment Scale, Activity Restriction Questionnaire, Carolinas Comfort Scale [26], Core Outcome Measures Index, Danish Hernia Database Questionnaire, Functional Ability Test, European Registry for Abdominal Wall Hernias Quality of Life Instrument, Functional Index Score, Hernia-specific Quality of Life Assessment Instrument, Inguinal Pain Questionnaire [27], McGill Pain Questionnaire, Pain Impact Questionnaire, and various SF versions. Validated instruments are rarely used, however, according to a recent critical review, which also reported that even among studies using them, assessments have not been recorded both before and after the procedures [28]. We suggest that validated questionnaires should be used systematically by both caregivers and researchers if we are to be able to understand the impact of CPSP on QoL in this and other surgical settings.

Our finding that 24.9% of patients with CPSP were taking some type of analgesic 4 months after surgery, and that the proportion had changed little after 2 years, is noteworthy. Kailliomaki et al.^7^ reported a much lower rate of analgesic use (2%), but they they did not look at how much time had passed since the patients’ operations. It is striking, however, that our review of the literature on recovery from groin hernia repair found that analgesic use is not systematically reported or analyzed in detail. The drugs most often taken by our patients with CPSP were nonsteroidal anti-inflammatory drugs, and there was little use of opiods or coadjutants. This last finding was particularly interesting given the proportion of CPSP patients with neuropathic pain (38.5%). We also observed that the percentages of patients with moderate/intense pain (46.4 to 52.1%, depending on the moment reported) and those taking analgesics (only 24.9 to 28.2%) were quite different. Moreover, only 30% of treated patients felt their analgesic regimens were effective. These data suggest that CPSP seems to be under-diagnosed and under-treated in routine clinical practice, at least in our mixed urban-rural area of eastern Spain. The incidence of CPSP we detected (13.6% at 4 months) was similar to results reported elsewhere [4, 5, 7, 29], but much lower than some reports of up to 60% [4–6]. Such variability can be attributed to diverse causes. The first is the very definition of CPSP, twenty-two of which have been used in studies on hernia repair alone according to Molegraaf et al*,*[28] who found that no definition at all had been made explicit in 39% of the studies reviewed. We used the definition of the International Association for the Study of Pain modified by Macrae and Davies [14], which is the one most often used across surgical settings. A second reason for variability is that many incidence rates are based on data derived only from postal or telephone surveys, without subsequent physical examination. The lack of an examining physician’s confirming diagnosis could lead to false positives, explaining higher incidence rates. The third reason for variability is that retrospective studies in the literature have used different periods of postsurgical follow-up.

The CPSP rates associated with mesh (13.8%) and non-mesh (4.4%) were dissimilar in our cohort but not significantly different, although we note that the number of non-mesh repairs was low (68 cases) and the number of confirmed CPSP cases was fewer than expected (3 rather than the 9 cases we expected). Still, this observation of nonsignificance and the similar rates of neuropathic pain after mesh and non-mesh repair suggest that using a mesh seems not to play a role in neuropathic CPSP. Rather, our observations suggest that the patient-related factors that form part of the GENDOLCAT group’s risk model (particularly young age, prior pain in the surgical field or elsewhere, and psychological profile) are the main predictors of both neuropathic and non-neuropathic CPSP after hernia repair, as they are after other procedures [1]. The rates of CPSP in both mesh and non-mesh repairs in this study are consistent with rates reported for other surgical settings in which mesh is not used, such as thoracotomies, breast surgery, and abdominal hysterectomies [1, 2]. The relatively low rate of neuropathic pain we observed also suggests that better medical rather than surgical approaches for preventing and treating pain are called for. We believe this is particularly true if we take into consideration that symptoms tended to resolve for about two-thirds of our patients with CPSP (affecting 6.2% after 1 year and 4.0% after 2 years), possibly attributable to the rarity of structural damage to nerves in the surgical field. However, while this might initially appear to point to the spontaneous resolution or attenuation of pain, we must emphasise that the percentage of CPSP patients with moderate or intense pain remained fairly constant (at 46.4% at 4 months to 51.9 and 52.1% at 1 and 2 years, respectively). This constancy may be explained by the under-treatment of CPSP we discuss above. Few other studies have analysed CPSP prospectively and long-term [7, 8].

An important strength of this study, which is the largest prospective long-term analysis of CPSP after groin hernia repair thus far, was its reliance on a trained, expert physical examination to confirm the CPSP diagnosis. Although one other recent study did include physical examination [21], the authors used a single anaesthetic technique in all operations and excluded patients with diabetes, prior neuropathic pain or ischaemic disease, limiting generalisability to the general surgical population. A second strength was our representative sampling of a previously characterised surgical caseload [30], such that the findings can be cautiously generalised to natural clinical settings. A final strength was the 2-year period of follow-up of patients with confirmed diagnoses, which we believe provides a reliable clinical picture of the long-term natural history of CPSP.

One limitation is that the questionnaires we used to identify QoL issues (the BPI and SF-12) were not specific to hernia repair. We did not use the Inguinal Pain Questionnaire [27] or the Carolinas Comfort Scale [31] because our data were extracted from the aforementioned larger study of a wider surgical population [1]. The two instruments we chose, however, have been validated in various surgical settings [31, 32] and have been adopted in other studies of CPSP after hernia repair [23–25]. Second, by classifying patients unavailable for phone screening at 3 months (13.7% of those enrolled) as not having pain, the CPSP incidence we report may be an underestimate. If we had simply eliminated these patients, the percentage of possible CPSP cases for examination would have been 15.9%, not 13.6%. The researchers made this decision in the interest of minimizing the number of false positives identified by the model and increasing the sensitivity. Our aim in modelling risk was to provide guidance for planning preventive measures or designing trials to confirm their efficacy. We wished to avoid treating patients who would receive no benefit from therapies. Third, given that only about a third of the patients reporting no pain at 3 months returned the SF-12 questionnaire by post, the results should interpreted cautiously when applying them to patients without diagnosed CPSP, whose situation may not be reliably reflected by our data. Fourth, the small number of non-mesh repairs in the cohort (68 patients, 3.87% of the total) may be the reason we did not detect significant differenes in CPSP between mesh and non-mesh repairs. Although statistical conditions for the analysis were met [33], we would like to see data for a larger number of non-mesh cases before arguing strong conclusions given that the CPSP rates between the two groups were quite different (4.4% vs 13.9%) and approached significance. A cohort with a larger number of non-mesh cases might have yielded different results. Finally, we did not do specific neurophysiological studies in either the CPSP patients at 4 months or in patients without CPSP after surgery. Our decision was based on reports that standardised tests often show persistent postoperative sensory dysfunction in patients without pain [34, 35]. Moreover, material and trained staff were not available in all of the 23 hospitals participating in the study.

## Conclusions

In conclusion, we have provided prospectively gathered data detailing the clinical features and management of CPSP at 4 months in the 13.6% of patients the GENDOLCAT [1] study identified with this complication of hernia repair surgery. Furthermore, we showed that pain persists in a sizeable proportion (about 4% at 2 years) of patients and may be undertreated or ineffectively treated. Our design attempted to respond to shortcomings in the literature on CPSP after hernia repair that have emerged as a result of variation in the definition of outcomes and the uncertain reliability and comparability of tools used for both the diagnosis and evaluation of CPSP’s impact on QoL.

## Supplementary information

**Additional file 1.** Online Resource 1: Data collection, variables, definitionsand telephone questionnaires.

## Data Availability

The datasets generated and/or analysed during the current study are not publicly available because we are involved in other studies with them. However, the corresponding author will make the data available in response to reasonable requests.

## Abbreviations

CPSP: Chronic postsurgical pain
QoL: Quality of life
HADS: Hospital Anxiety and Depression Scale
SF-12: The Short Form Health Survey-12 questionnaire
BPI: The Brief Pain Inventory
DN4: Douleur Neuropathique 4
SF-36: The 36-Item Short Form Health Survey
