# Supplementary material for: Chronic pain after groin hernia repair: pain characteristics and impact on quality of life
Source: BMC Surg. 2020 Jul 6;20:147. doi: 10.1186/s12893-020-00805-9 (PMC7336434; doi:10.1186/s12893-020-00805-9)
Supplement: Supplementary file 1 — Additional file 1. Online Resource 1: Data collection, variables, definitionsand telephone questionnaires. [file 12893_2020_805_MOESM1_ESM.doc]

**Data collection questionnaires: variables and definitions**

**Variables recorded on enrollment**

- Diagnosis leading to surgery
- Description of surgical procedure
- Type of surgery: scheduled or emergency
- Sex
- Age in years
- Place of birth (Spanish autonomous community)
- Date of surgery
- Employment status
- Education in years of formal schooling

**Variables recorded in the preoperative assessment visit with anesthesiologist**

- Weight and height
- Alcohol intake: positive if >24 g/d (>3 glasses of wine, or >3 beers or ≥2 glasses of hard liquor)
- Smoking:
  - No
  - Current
  - Ex-smoker (>3 months since last smoke)
- Chronic obstructive pulmonary disease: answer “yes” or “no” to the question “Has any physician told you that you have a chronic respiratory disease?”
- Heart disease declared by the patient, stable or unstable
- Hypertension: answer “yes” if the patient reports this diagnosis or is on an antihypertensive drug
- Peripheral circulatory disease declared by the patient or recorded in the chart
- Neurologic disease declared by the patient or recorded in the chart
- Chronic kidney failure with or without dialysis
- Liver disease declared by the patient, recorded in the chart, or observed (previous jaundice, hepatomegaly or ascites)
- Diabetes mellitus: treated with dietary measures, with oral antidiabetic therapy alone, insulin alone, or an oral antidiabetic agent plus insulin
- Cancer: Karnofsky score >50% or ≤50%
- Immunosuppression declared by patient (treatment) or disease recorded in chart
- American Society of Anesthesiologists physical status classification (1-4)

**Preoperative pain assessment**

- Drugs used for reasons other than pain
  - Benzodiazepines
  - Anticonvulsants: ongoing treatment or only preoperative
  - Antidepressants
  - Corticosteroids
  - Street drugs (cannabis, cocaine, heroine, etc): If cannabis is being used for therapeutic purposes, record “no” or if ≤2 cigarettes/d are smoked
- Presence of pain at the site of the intervention or other parts of the body during the 24 hours prior to surgery, with intensity expressed on a verbal numerical rating scale (VNRS) (0=no pain, 10=the worst imaginable pain to 10) (Values > 3 were considered positive in the bivariable and multivariable analyses)^1^
- Use of analgesics or other agents for pain: paracetamol, nonsteroidal anti-inflammatory drugs, metamizole, low-dose opioids, strong opioids, antidepressants, anticonvulsants
- Previous experience of intense postoperative pain
- Family history of chronic postoperative pain: grandparents, parents, siblings, children
- Short Form Health Survey (SF-12), for quality of life (version 2; time frame, 4 weeks) (Spanish version)^2^
- Hospital Anxiety and Depression Scale (Spanish version).^3^ The 14 items are scored on a 4-point Likert scale. Seven items measuring anxiety and seven measuring depression over the last week provide a total score of between 0 and 21 for each subscale. The cut-off point for anxiety or depression is 8

**Intraoperative variables**

- Surgical incision
  - Direct inguinal hernia repair:
    - Anterior inguinal repair, Liechtenstein mesh hernioplasty
    - Anterior preperitoneal repair, Nyhus or Stoppa technique
    - Repair with plugs (Rutkow/Rutkow-Robbins/Gilbert)
  - Femoral hernia repair:
    - Open repair
    - Repair with plugs (Ruklow/Rutkow-Robbins/Gilbert)
    - Meshless repair with suture anchoring (McVay technique)
- Nerve-sparing (only in inguinal hernia repair)
- Type of anesthesia (general, including combined with regional anesthesia, or spinal, epidural, plexus nerve block, local)
- Drains
- Opioid doses (fentanyl, remifentanil, alfentanil, morphine)
- Corticosteroids
- Ondansetron
- Ketamine
- Level of sensory blockade
- Spinal opioid doses (morphine, fentanyl, methadone)
- Type of local anesthetic injected spinally: bupivacaine, mepivacaine, lidocaine, ropivacaine, levobupivacaine, prilocaine
- Results of spinal anesthesia: good, moderate, poor
- Blood loss, in milliliters
- Duration of surgery in minutes (from skin incision to skin closure)
- Destination on discharge from the surgical area: home (major ambulatory surgery), hospital ward (via recovery room), critical care unit, exitus

**Postoperative variables**

- Pain 24 hours after surgery (VNRS)
- Postoperative length of stay (number of days from the intervention day to discharge)
- Analgesics (oral or parenteral): paracetamol, NSAIDs, metamizole, opioids, tramadol, ketamine, neuroleptics, gabapentinoids
- Infusion and infiltration techniques to manage postoperative pain: epidural or paravertebral infusion, wound infusion, peripheral nerve block
- Surgical wound complications during hospitalization (abscess, fistula, dehiscence, seroma, hematoma)
- Reoperation, using a different incision

**Variables recorded at the follow-up visit between 3.5 and 4.5 months**

- Date of visit
- Anatomical site of pain
- Brief Pain Inventory (BPI-Spanish version)^4^ (Values > 3 on a scale of 0 to 10 were considered positive indications of interference caused by pain)
- Neuropathic Pain questionnaire (version 4, Douleur Neuropathique 4 [DN4]) (Spanish version)^5^ (cut-off point of 4 out of 10)
- SF-12 questionnaire (version 2, time frame of 4 weeks (Spanish version)
- Analgesic treatments used: paracetamol, non-steroidal anti-inflammatory drug, metamizole, weak opioids, strong opioids, paracetamol-tramadol combination, anticonvulsants, antidepressants, topical analgesic, nerve block, other

**Questionnaire for telephone interviews (between 1 and 1.5 months, 2.5 and 3.5 months, at 12 months and at 24 months)**

Note the number of days since the operation before the interview.

Ask the patient:

- Do you have (or continue to have) pain related to the operation?

If yes, ask the following questions

- Have you had any problems with the incision?
- Have you had radiotherapy or chemotherapy?
- Have you had to have another operation for any reason, an operation different from the one you had when you joined this study?
- Is the pain you have now like the pain you had after the operation?
- Is the pain continuous or does it come and go?
- If the pain is intermittent, how long does it last? Seconds? Minutes? Hours? Days?
- How bad is the pain on a scale of 0 (meaning no pain at all) to 10 (the worst pain you can imagine)?
- What do you take for your pain?

Allow the patient to express the medication names freely, by active principles or brand names. Classify the replies into the following categories:

- Paracetamol
- Nonsteroidal antiinflammatory drugs
- Metamizole
- Weak opioids
- Strong opioids
- Paracetamol and tramadol in combination
- Anticonvulsant
- Antidepressant
- Topical treatment
- Nerve block
- Other
- Would you say the treatment you’re using is effective in relieving your pain? (Not at all? A bit? Somewhat? Pretty well? Very well?)
- Are you still employed or working on your own?

If yes, ask:

- Have you returned to work yet?

Results of the telephone call:

- Exitus
- The patient could not be reached.
- The patient was reached but did not adequately complete the interview.
- The patient was reached and successfully interviewed.

**References**

1. Allvin R, Brasseur L, Crul B, et al. European Minimum Standards for the Management of Postoperative Pain. EuroPain Task Force. Pegasus Healthcare Intl, UK, 1998.
2. Schmidt S, Vilagut G, Garin O et al. Reference guidelines for the 12-Item Short-Form Health Survey version 2 based on the Catalan general population. Med Clin (Barc) 2012;139(14):613-25.
3. Caro I, Ibañez E. La escala hospitalaria de ansiedad y depresión. Su utilidad práctica en psicología de la salud. Boletín de Psicología 1992;36:43-69.
4. Badia X, Muriel C, Gracia A, et al. Validation of the Spanish versión of Brief Pain Inventory in patients with oncological pain. Med Clin (Barc) 2003;120:52-9.
5. Pérez C, Galvez R, Huelbes S, et al. Validity and reliability of the Spanish version of the DN4 (Doleur Neuropathique 4 questions) questionnaire for differential diagnosis of pain syndromes associated to a neuropathic or somatic component. Health Qual Life Outcomes 2007;5:66.
